# A Consistent Approach to Modeling Quantum Observers

**DOI:** 10.3390/e27030302

**Published:** 2025-03-14

**Authors:** David W. Ring

**Affiliations:** Independent Researcher, Longmont, CO 80503, USA; physicspapers@davering.com

**Keywords:** quantum mechanics, quantum measurement, quantum foundations, quantum computers, quantum circuits

## Abstract

A number of no-go theorems have shown that Wigner’s Friend scenarios combined with various metaphysical assumptions lead to contradictions in any version of quantum theory. We present an alternative constructive approach that only assumes that agents make properly qualified true statements. Quantum observers are modeled rigorously, although simplistically, using quantum circuits. Terminology is suggested to help avoid contradictions. Our methodology is applied to the Frauchiger-Renner paradox and results in statements by all agents that are both true and consistent. Quantum theory evades the no-go theorems because they make an incorrect implicit assumption about how quantum agents behave.

## 1. Introduction

Quantum theory challenges our intuition in many ways. Issues around observation are especially controversial. Observers are physical entities and presumably should obey the same rules as non-thinking objects. Observers must, therefore, be able to have multiple thoughts in superposition, at least in principle. Indeed, today’s quantum computers are able to process bits of information that are in superposition. Quantum computers are universal computing machines, and there is no limitation, in principle, to the complexity of quantum circuits, so it is reasonable to assume that a quantum observer’s thoughts could, in fact, exist in superposition.

This is particularly relevant to Wigner’s Friend (WF) scenarios, where observers are part of the experiment and are treated quantum mechanically. Wigner proposed a thought experiment [1] where a quantum system is observed by agent F, who is, herself, observed by agent W. Following the Copenhagen interpretation [2], W and F make contradictory statements about the resulting quantum state. Many conceptual analyses have been performed on such scenarios, often in the context of other interpretations.

Interest in the topic was renewed recently when an extended version of WF was developed by Frauchiger and Renner (FR) [3]. In this version, four agents interact with a quantum system and reason about each other’s thoughts. FR proved that no quantum theory can be consistent with a set of reasonable-sounding assumptions. Naturally, the paper was controversial, with many authors criticizing it [4,5] or defending their preferred interpretation [6,7,8,9].

Following this, a number of authors [10,11,12,13,14,15,16] have proved remarkable no-go theorems that state that no interpretation of quantum theory can be consistent with various sets of metaphysical assumptions. Therefore, either quantum theory is wrong, or one or more of the assumptions is wrong. These no-go theorems are considered the state of the art in this area.

Consider Theorem 1 from reference [12]: “If a superobserver can perform arbitrary quantum operations on an observer and its environment, then no physical theory can satisfy Local Friendliness”. Here, Local Friendliness (LF) is one specific combination of metaphysical assumptions. It is natural, then, to consider an observer that is an AI program running on a quantum computer. We can, in fact, perform arbitrary quantum operations on the qbits in quantum computers today, but only for simple models. Since there are no barriers, in principle, to scaling quantum computers up to support AIs, it is of obvious interest to explore the theoretical capabilities and behavior of quantum circuits in the context of WF scenarios. Such an analysis has been carried out at a conceptual level [17] by assuming the AI satisfies a modified set of metaphysical assumptions designed to avoid the question of whether the AI actually performs a measurement.

The purpose of this paper is to explore an entirely different, constructive approach. Instead of debating the virtues of various metaphysical assumptions, quantum circuits are developed step by step under the sole assumption that the quantum agents they represent have super-human intelligence and, therefore, will not make false or inconsistent statements. Making a statement (or, equivalently, knowing something) when your thoughts are in superposition is tricky, and it will be helpful to carefully and rigorously define the meaning of the terminology used. Using quantum circuits to analyze thought experiments goes back at least to Deutsch and Hayden [18], who used them to quantify information flow in non-local entangled states. They are naturally useful as a standard and compact way to diagram unitary transformations of quantum information, similar to the way Feynman diagrams simplify field theory calculations.

We are not able to construct an entire circuit to implement artificial intelligence, but that will prove unnecessary. In scenarios where the observer has a simple knowledge goal, we can design simple circuits to prepare that knowledge and represent it with a single qbit or a small number of qbits. A general quantum AI operating reversibly would be able to do the same thing, and the algorithm itself is not of interest here, only the simultaneous relationship between the knowledge qbits.

The literature on this topic is vast, and we will not attempt to summarize all points of view. See [9,17] for many useful references.

The discussion is organized as follows. Section 2 clarifies how we describe quantum states after observation and how we avoid the measurement problem. Section 3 demonstrates a simple circuit modeling observation reversal, as in Deutsch’s seminal thought experiment [19]. Section 4 introduces *virtual knowledge* and related concepts in an intuitive way. Section 5 refines these concepts and defines terminology that will make our discussions more rigorous and may be used by the agents themselves as they make precise knowledge statements. Section 6 models simple scenarios where multiple agents reason about each other’s knowledge. Section 7 explicitly demonstrates simple cat measurements and briefly discusses the limitations of complementary observables. Section 8 briefly discusses retrospective and predictive knowledge. Section 9 applies the methodology to the FR paradox to ensure we can successfully model a more complex scenario and to demonstrate that agents making precise, properly qualified statements do not produce contradictions. Section 10 models a weak measurement in the context of a WF scenario. Section 11 describes how quantum theory evades the no-go theorems. Finally, Section 12 presents a fictional first-hand view of how a quantum agent might experience one of these experiments.

## 2. Observation States

The details of what happens during an observation are highly controversial, and the measurement problem has defied consensus for nearly a century. In this paper, we will remain agnostic on this issue and, instead, consider possible forms for post-observation states. We only assume that the laboratory, and indeed the universe, is describable before and after an observation by states, either pure or mixed. We do not consider how any particular interpretation would construe our results.

When an observation takes place, the combined state of the system, apparatus, and observer becomes correlated. In the case of a quantum observer, the three components may be tightly coupled and/or coherent, and the lines of separation between the components may be blurred. For our purposes, we will separate the observer’s memory and conclusions from the rest. We will call the former her *knowledge* and the latter the *context*. The context may include other observers and their knowledge.

We consider three types of post-observation states: entangled, siloed, and collapsed. The density operator for an entangled observation result may be written as

(1) $$\rho_{e}=\sum_{ab}\rho_{ab}⊗|a\rangle \langle b|\;$$

where $\rho_{ab}$ operates in the context Hilbert space, and *a* and *b* index the distinguishable results of the observation in the knowledge Hilbert space. For simplicity, we assume the knowledge basis is not degenerate. Even with off-diagonal terms, expression (1) may describe a product state where the knowledge state is not a basis vector. This, by definition, is not entangled. Nevertheless, we will analyze it as if it were an entangled state. The term *pseudo-entangled* may be used. In all of our thought experiments, the initial state will be pure, but that is not a requirement.

If, instead, the off-diagonal components are zero, we have a siloed correlation:

(2) $$\rho_{s}=\sum_{a}\rho_{a}⊗|a\rangle \langle a|\;.$$

This is a mixed state and may be considered an incoherent superposition. Evolving from a pure state to a mixed state requires loss of information. A loss of “which-way” information [20] is sufficient to guarantee that off-diagonal components are zero [21].

If quantum collapse occurs, a projection operator

(3) $$\Pi_{a}=I⊗|a\rangle \langle a|$$

is applied, and we have a collapsed state:

(4) $$\rho_{c}=N_{a}\rho_{a}⊗|a\rangle \langle a|\;,$$

where a normalization, $N_{a}$, is added because the projection operator changes the trace.

If an observation results in a siloed state or a collapsed state, we will call it a *measurement*. Otherwise, we call it an *observational entanglement*. $\rho_{s}$ and $\rho_{c}$ are equivalent under unitary evolution in the sense that no future experiment can distinguish them. There is no analogous equivalence between the unitary evolution of entangled states and collapsed states, so it is wrong to apply (3) to the former. Nevertheless, we will consider the possibility that some non-unitary physical processes can evolve an entangled observation result into a collapsed state. We will insist, however, that for each observation, whether measurement occurs or not is determined by the experimental setup.

In WF scenarios, each friend, F, and her laboratory are usually assumed to evolve unitarily so that the entire laboratory may be measured by the superobserver, W. This means the observation of F is necessarily an observational entanglement and not a measurement. Indeed, allowing F to complete a measurement (with or without the final collapse projection) would destroy the interference necessary to produce the desired contradictory outcome. Our simplified circuit models demonstrate explicitly that such a unitary evolution is possible. Whether macroscopic scale circuits, as may be required for general AI, can remain coherent is beyond the scope of this article, but see [17] for a discussion.

## 3. Remeasurement

Deutsch was the first to analyze quantum observers with some rigor. He proposed a thought experiment [19] that would have a different result in a unitary universe as opposed to a universe where collapse occurs. A spin is prepared pointing along the positive x-axis. It passes through a Stern Gerlach magnet aligned along the z-axis and an agent’s sense organ before being deposited in a storage ring. The agent records the completion of the observation but not which result was seen. This is reminiscent of the Wigner’s Friend argument, where the agent can be asked how she felt about her observation. Then, the reverse Hamiltonian is applied, pulling the spin out of the storage ring, erasing the agent’s knowledge, and possibly restoring the original prepared spin state. The completion record is somewhat of a red herring since it is (ideally) always set, and if the reverse Hamiltonian includes it, it will also be erased. In fact, the agent may have no way to know if the experiment has even been performed.

If the spin is then measured along the x-axis, the result will depend on whether collapse occurred. If collapse occurs, either result will happen. Otherwise, only the prepared state will be seen. Thus, the experiment can distinguish the two models.

**Figure 1:**
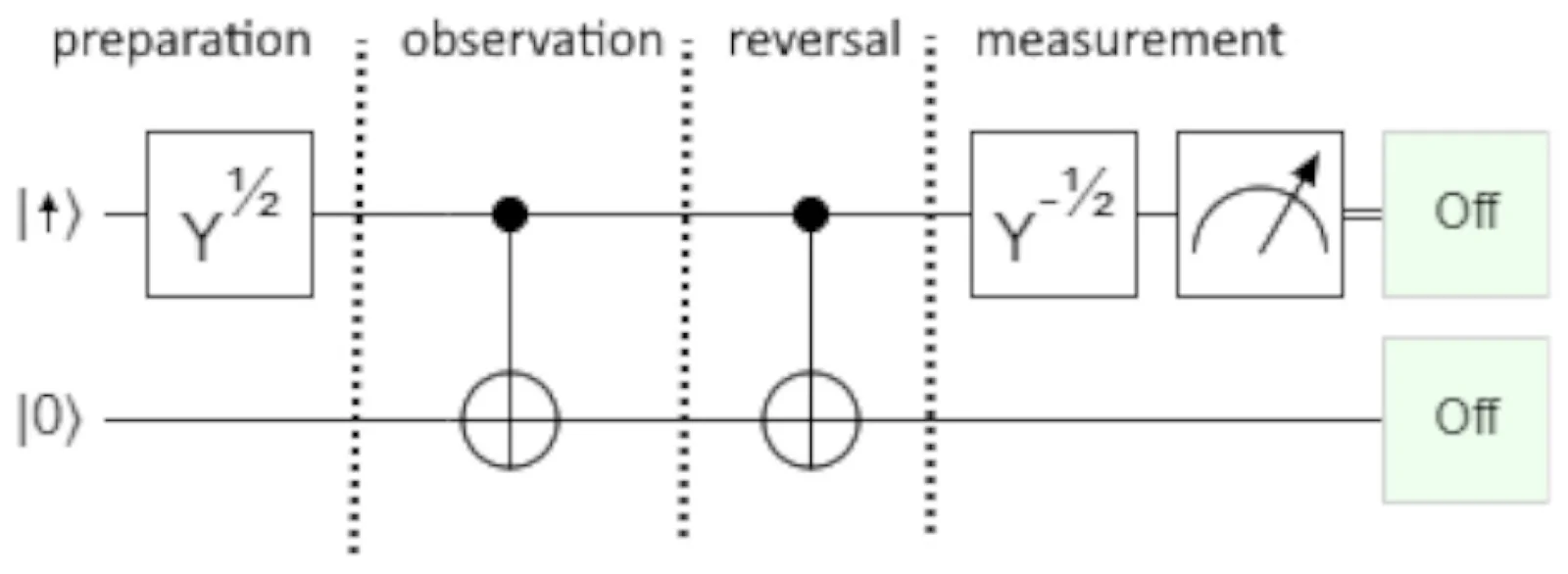
Reversal of an observation. The first line is a qbit representing a spin system. The second line represents the agent’s knowledge. The spin is observed, and the agent knows the spin is ‘up’ and also that it is ‘down’. The observation is reversed, erasing the agent’s knowledge and allowing a different measurement to be performed.

We can demonstrate this using the quantum circuit in Figure 1 (all figures in this paper were produced and simulated using Quirk v2.3: https://algassert.com/quirk). The first line is a qbit representing a spin system. A |0〉 state may be thought of as a spin pointing along the +z-axis, but we will usually represent spin system states with direction arrows and knowledge and computation qbits with 0 s and 1 s. In order to prepare the spin pointing along the +x-axis, a $Y^{1/2}$ gate is applied, rotating the spin by π/2 radians. The second line represents the agent’s knowledge. The spin is observed using a CNOT gate. The spin and the agent’s memory are now entangled in a state.

(5) $$|\psi \rangle =\left(|↑0\rangle +|↓1\rangle \right)/\sqrt{2}\;.$$

At this point, we could say the agent has knowledge of the spin’s z-component, but the knowledge is different in the two components of the superposition. We call this *virtual* knowledge.

The observation can be reversed using a second CNOT gate. This erases the agent’s virtual knowledge and restores the prepared spin state (|→〉). We can then measure the spin’s x component by rotating it with a $Y^{-1/2}$ gate and applying a measurement gate. In Figure 1, the measurement is successful: “off” (=|0〉) implies the spin was prepared as |→〉. For this quantum circuit, at least, observational entanglement does not provoke collapse. There is no reason to believe Deutsch’s experiment would behave any differently. Given the ease with which quantum circuits can be constructed and tested today, we should feel confident that *observational entanglement alone is not sufficient to cause collapse*. The author considers this prediction and verification to be one of the most significant and underappreciated results in the last half-century of physics.

## 4. Virtual Knowledge

We could allow our agents to parse and emit English sentences encoded in classical bits or qbits that they know will be either 0 or 1. However, it is generally simpler to represent statements with a single qbit. This is similar in spirit to the controversial “grandmother cells” in neuroscience [22]. The meaning of the qbit is encoded in reasoning circuits that we do not attempt to model here. The details do not affect our results because the reversal of unitary circuits is straightforward. In the observation step of Figure 1, the agent could set her memory to |1〉 to represent the statement “in my branch the spin is down”. This statement applies to the component/branch of the superposition state in which the agent is making that statement. The qualification “in my branch” is necessary. Without it, the statement would be false. The spin is not, in fact, in a ‘down’ state.

The meaning of the qualification is relatively clear here, but later, we will find it insufficiently rigorous. We, therefore, define a branch as a synonym for a *knowledge projection*. Starting from the general state (1), the knowledge projection is given by acting with the projector (3), giving (4). Note that the knowledge projection after a single entanglement observation is identical to the state that would have occurred if the observation was, instead, a measurement with associated collapse.

Bear in mind that quantum logic processing is, in general, designed around a particular basis, often called the computation basis. As an example, the entangled state in Figure 1, $\left(|↑↑\rangle +|↓↓\rangle \right)/\sqrt{2}$ can also be written $\left(|\to \to \rangle +|\leftarrow \leftarrow \rangle \right)/\sqrt{2}$. Consider an English language multi-qbit virtual knowledge register deduced by the agent. The register holds a five-character string with possible values “___up”, “_down”, “_left”, and “right”. If, in one basis, the last character is absolutely ‘t’, it cannot be ‘p’ or ‘n’ in another basis.

## 5. Terminology

When discussing quantum observers, we will use the following terminology:

*Entanglement*—An entangled correlation. A condition/evolution where an agent is/becomes coherently correlated with a system. If the state is a product state, the term *pseudo-entangled* may be used.
*Measurement*—An evolution where an agent becomes incoherently correlated with a system. If a collapse projection is applied, we can say that only one result “actually” occurred.
*Observation*—An evolution that is either entanglement or measurement.
*Knowledge projection*—The state formed by projecting to a particular knowledge state. This is also called a *branch*.
*Absolute knowledge*—Information that is true about the actual physical state.
*Virtual knowledge*—Information that is true in its own branch but not in others.

Note that “absolute” and “virtual” do not distinguish whether the information is *known* to all branches. Instead, they describe whether the information is *true* in all branches. A single agent can have both virtual and absolute knowledge. For example, the agent in the Deutsch experiment may know that the spin she has been given is an electron. This is defined as absolute knowledge because it is true for the entire physical state. She also knows its z-axis component. This is virtual knowledge because the value is different in different branches. In general, we can read off the type of knowledge from the entangled system/observer state. For example, state (5) can be extended to include a qbit representing the observer’s knowledge that the spin is an electron:

(6) $$|\psi \rangle =\left(|↑01\rangle +|↓11\rangle \right)/\sqrt{2}.$$

The new qbit is 1 for the entire state, so it is absolute knowledge, while the knowledge of the z-axis component is different in different components of the superposition, so it is virtual knowledge. Similar considerations apply to a mixed state.

As we will see, there are even scenarios where an observer can have absolute knowledge in one branch but not another.

Note that in all of our thought experiments, there is an implicit experimenter sitting outside the laboratory. The reader may imagine that to be themself. This experimenter is the ultimate arbiter. His knowledge is always absolute. All our state notations are written from the perspective of this experimenter.

If the procedures for an experiment and the initial pure state are provided to the agents, as we will usually assume, then the entire evolution of the quantum state will be available for deduction as absolute knowledge, including other agents’ virtual thoughts, but only up to the moment of any measurement. Explicit recursive reasoning, such as “I know that I know...” or “I know that she knows that I know...”, is limited by available memory.

If the experimental details are not provided to the agents, they will have uncertainty and limited knowledge. In particular, they may draw wrong conclusions if they do not know they are in superposition.

## 6. Multiple Agents

Given that agents can store and process virtual knowledge, it should be possible for them to reason about other agents. We will construct explicit examples using quantum circuits. We will not attempt to develop general intelligence but rather find simple circuits that solve particular problems.

**Figure 2:**
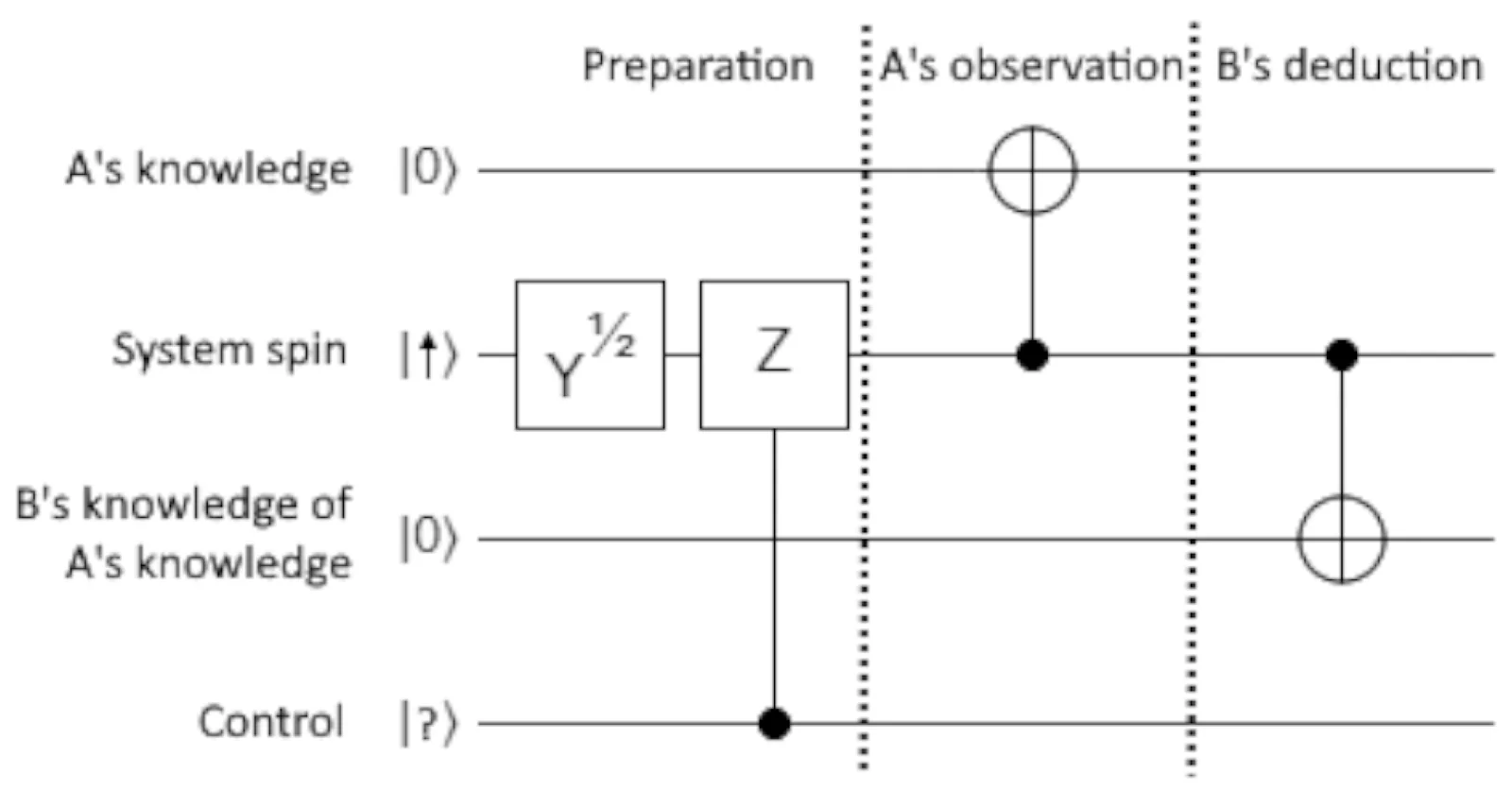
Simple virtual deduction. The experimenter uses a control bit to prepare a system spin. Alice observes the system and records its z-axis values in a qbit register as virtual knowledge. Bob inspects the system to determine Alice’s virtual knowledge. He is able to correctly deduce virtual knowledge of Alice’s virtual knowledge even though neither agent knows whether the Z gate has been applied.

Figure 2 models a spin system and two agents. The spin is prepared based on a control bit that is set to 0 or 1 in each run by the experimenter. The prepared spin is in a state |→〉 or |←〉, depending on whether the Z gate is applied. The agents do not know which state is prepared. The first agent, Alice, interacts with the system and gains virtual knowledge that the spin is ‘up’ and also that it is ‘down’. The second agent, Bob, interacts with the system in order to deduce virtual knowledge that Alice saw ‘up’ and that she saw ‘down’. The final state is one of

(7) $$\begin{array}{cc} \; & |R\rangle =\left(|↑00\rangle +|↓11\rangle \right)/\sqrt{2}\\ \; & |L\rangle =\left(|↑00\rangle -|↓11\rangle \right)/\sqrt{2} \end{array}$$

where the states on the left are labeled by the x component of the original prepared spin, and each ket on the right is labeled by (1) the z component of the system spin, (2) Alice’s virtual knowledge (e.g., 1 means the system spin is ‘down’), and (3) Bob’s knowledge of Alice’s knowledge. We can read off that all the virtual knowledge is valid since each ket is fully aligned regardless of which spin state was prepared.

**Figure 3:**
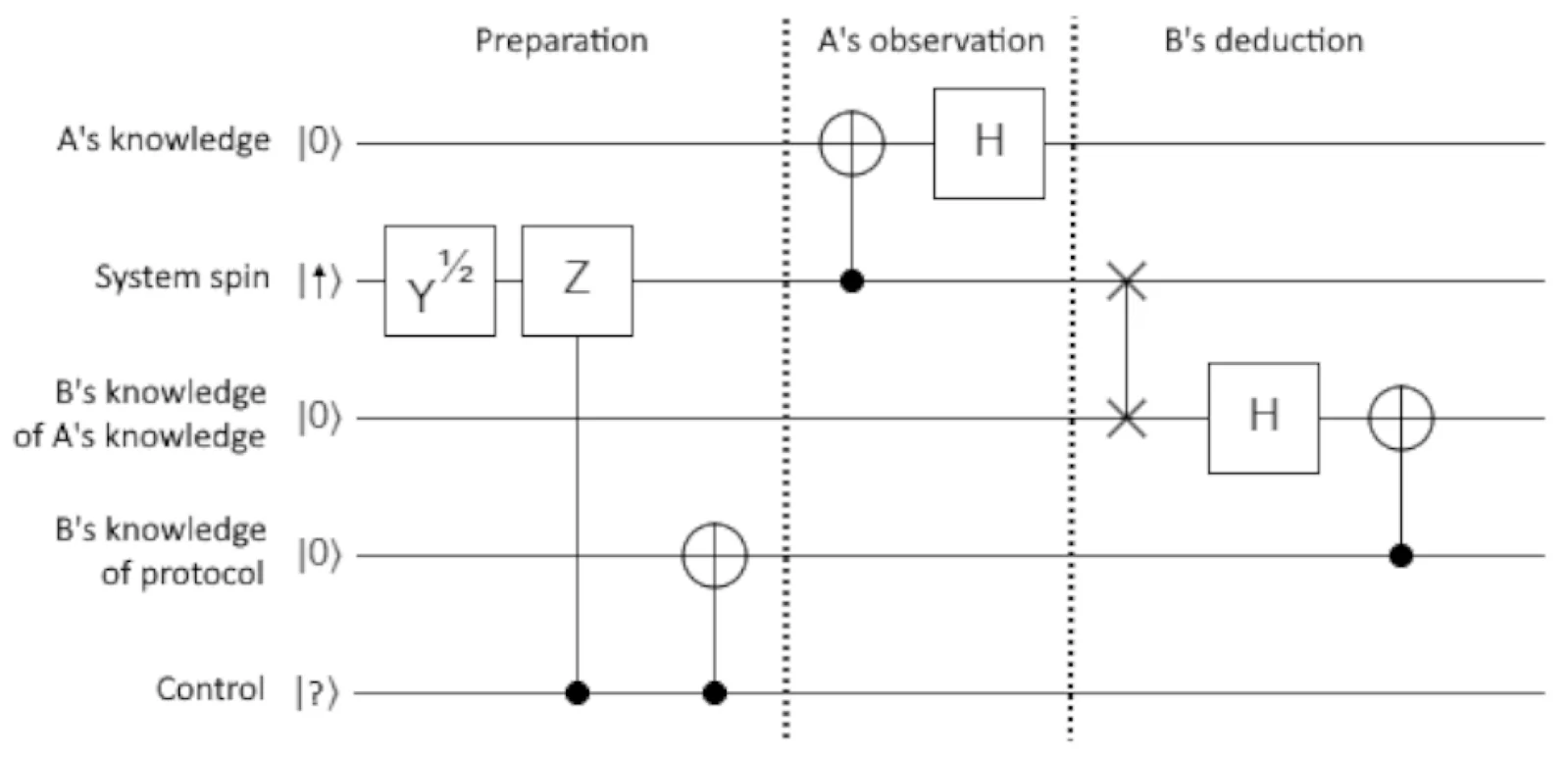
Virtual deduction with protocol knowledge. The experimenter prepares the spin and informs Bob using a CNOT gate. Alice processes her observation before recording her virtual knowledge. Bob is able to deduce her virtual knowledge using absolute knowledge of the experimental protocol. Without protocol knowledge, it is impossible for Bob to deduce Alice’s knowledge.

In Figure 3, Alice tries to prevent Bob from learning her knowledge. She applies a Hadamard gate before recording her observation. Bob can still perform the deduction if he has information about the experimental protocol. He first interacts with the system using a swap gate. Note that this is a destructive process; the system is left in a fixed reference state, |↑〉. He then applies his own Hadamard. Finally, he applies a CNOT gate to flip his virtual knowledge in the case where he knows the Z gate was applied. The final state is

(8) $$|R^{\prime }\rangle =|L^{\prime }\rangle =\left(|↑00\rangle -|↑11\rangle \right)/\sqrt{2}\;.$$

Bob has successfully deduced Alice’s knowledge.

Without the extra protocol knowledge, it is impossible for Bob to deduce Alice’s knowledge. The proof is as follows. Consider the state after Alice’s observation and Hadamard but before Bob’s deduction. Using the same three-part notation as above, it is one of

(9) $$\begin{array}{cc} \; & |R\rangle =\left(|\leftarrow 10\rangle +|\to 00\rangle \right)/\sqrt{2}\\ \; & |L\rangle =\left(|\leftarrow 00\rangle +|\to 10\rangle \right)/\sqrt{2}\;. \end{array}$$

These states can be swapped by applying a NOT gate to Alice’s knowledge. If Bob manages to make his virtual knowledge aligned with Alice’s in the case that Z is not applied, his knowledge would be exactly wrong in the case where Z is applied. Whatever logic circuitry he used to make his deduction would be incorrect.

These circuits are deliberately trivial. A real quantum AI would perform the observation but would have more complex circuitry. The knowledge register would need more qbits to provide context and to distinguish a register of value 0 from an unset register. Knowledge might be represented more abstractly, for example, by phases rather than simple register values. Nevertheless, a quantum computer is a universal computer, and if it is logically possible for us to deduce virtual knowledge from information available to an agent, then a circuit can be designed to perform the same deduction. In the previous example, it is not logically possible for Bob to deduce Alice’s knowledge from supplied information since an experimenter’s choice can invalidate any such knowledge.

## 7. Cat Measurements and Complementarity

The combination of reversal and measurement in Figure 1 is an example of what is sometimes called a “cat measurement”. The name comes from the idea of proving Schrödinger’s cat is in a superposition by measuring the entire cat coherently. In our case, the experimenter measures the entire laboratory, including the system and observer.

**Figure 4:**
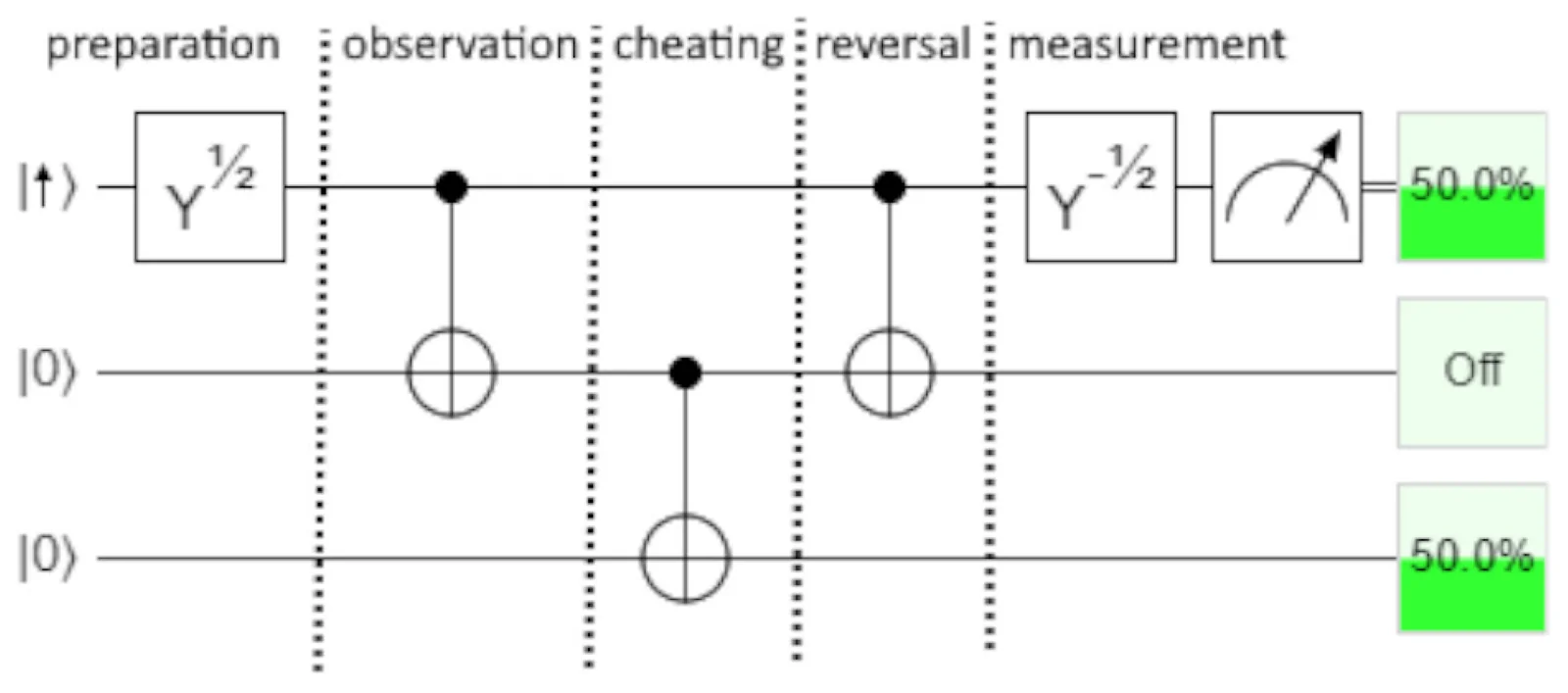
Attempted cheating. If we try to cheat and copy the z-axis observation result before the reversal, the x-axis measurement fails. Note the green boxes provide a graphical depiction of final probabilities.

Cat measurements are often used in thought experiments to measure in a complementary basis. This requires the observer’s memory to be erased. Figure 4 represents an attempt by a second observer to cheat and find out both the x-component and z-component of the prepared spin. An additional CNOT gate is used to copy the observation result before reversing the observation. In this case, the measurement fails: either result is possible.

**Figure 5:**
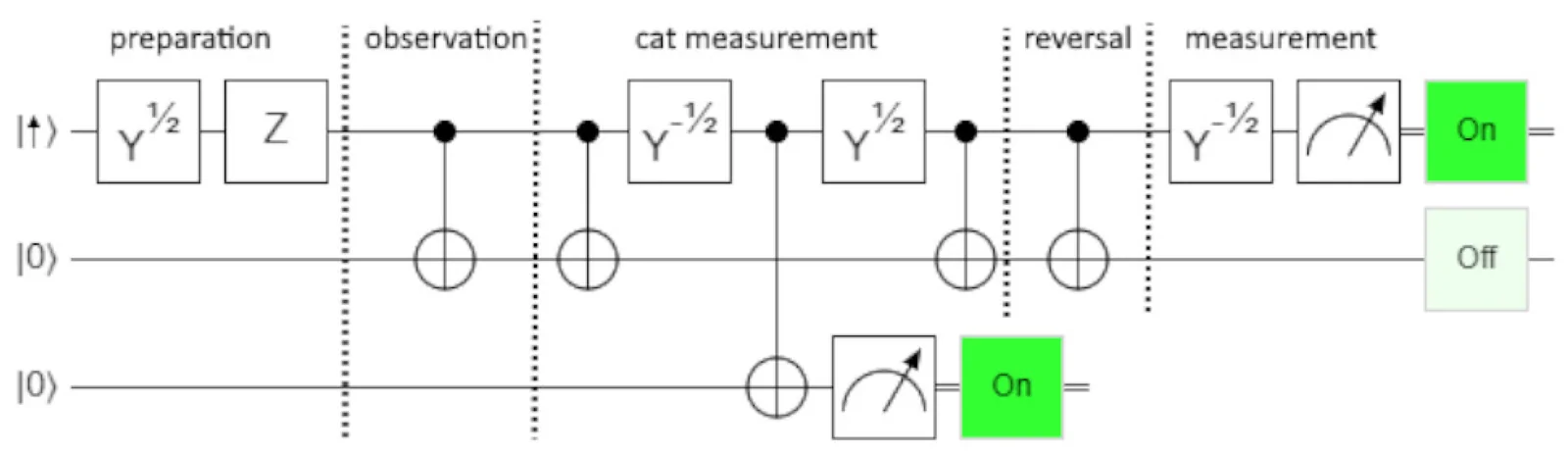
Not cheating. It is possible to “cat measure” in a basis consistent with another measurement.

It matters what information is copied. In Figure 5, the second agent performs a cat measurement to learn what the x-axis measurement result will be. Both measurements result in “on”, meaning the spin was prepared, pointing along the -x-axis. If the Z gate is removed, both measurements will result in “off”, meaning the spin was prepared pointing along the +x-axis.

## 8. Retrospections and Predictions

Consider the “reversal” circuit in Figure 1. Between the two CNOT gates, the agent is in a superposition of knowing the system spin is up and knowing it is down. This is virtual knowledge. If the agent wrongly treats her knowledge as absolute, she may deduce, in each branch, statement S: “the x-axis measurement has a 50% chance of being −¯h/2” (this would show as “50.0%” in the circuit diagram). A correct virtual knowledge statement might be “in my branch, S is true”. In reality, the two components of the superposition interfere, and that value never occurs. This is important enough to deserve a name. We will call it the *virtual promotion fallacy*: virtual knowledge is wrongly promoted to absolute. In this case, S is true in all branches, and yet S is absolutely false. Virtual knowledge cannot be trusted, especially when it comes to measurement predictions.

On the other hand, if the first $Y^{1/2}$ gate is optional (decided in each run by the experimenter), it is possible for the agent to deduce in one of the two branches that the gate has been applied. This is absolute retrospective knowledge that is only available in one branch, and its statement does not need the branch qualification. That agent-branch can then be certain the measurement will be “off”. This is a special case where a quantum agent with partial knowledge is able to make an absolute measurement prediction. Bear in mind that this example assumes the unitary operations comprising the agent’s logic will be reversed and the conclusion forgotten before the second CNOT gate is applied. Otherwise, we need to apply something like the “cheating” circuit.

## 9. Frauchiger and Renner

The title of Frauchiger and Renner’s paper is “quantum theory cannot consistently describe the use of itself”. We accept this as a challenge. The scenario is somewhat intricate, and we will not reproduce all of it here. We recommend the reader have a copy of their paper handy. The experiment involves four agents. The observations of two of the agents, $\bar{F}$ and *F*, are entanglements rather than measurements. Agents $\bar{W}$ and *W* perform cat measurements. All agents are aware of the experimental protocol, including the fact that the “coin flip” is a superposition.

The relevant states are listed in Table 1. The experiment proceeds as follows:

The state is prepared as $|\psi_{0}\rangle$.

$\bar{F}$ observes the “coin”. If *h* is observed, she sets the spin to ↓. Otherwise, she sets the spin to →, leading to $|\psi_{1}\rangle$. In the latter case, she also flips her knowledge qbit to indicate her belief in statement $S_{\bar{F}}$, leading to $|\psi_{2}\rangle$. Note that the result would be the same if the knowledge qbit was set first and used to set the spin.

*F* observes the spin. If it is ‘up’, she flips her knowledge qbit to indicate her belief in statement $S_{F}$, leading to $|\psi_{3}\rangle$.

$\bar{W}$ cat measures $\bar{F}$. On some runs, he obtains the result $\bar{ok}$, leading to state $|\psi_{4}\rangle$.

*W* cat measures *F*. On some runs, he obtains the result ok, leading to state $|\psi_{5}\rangle$.

**Figure 6:**
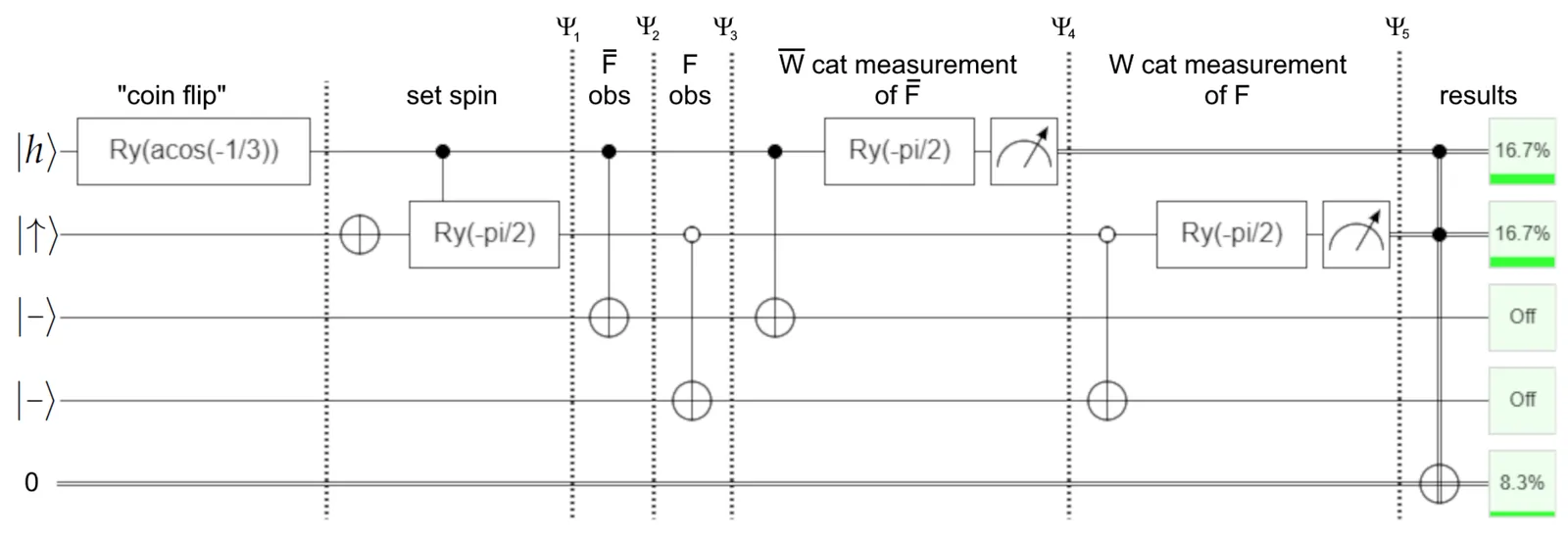
The FR experiment (doubled). The four qbits are h/t, ↑/↓, -/$S_{\bar{F}}$, and -/$S_{F}$, and each of these pairs is identified with 0/1. Rotation gates are used because a controlled $Y^{-1/2}$ introduces an undesirable phase. After the cat measurements, the first two qbits are repurposed for measurement results $\bar{fail}/\bar{ok}$ and fail/ok, respectively. The final classical bit will be true if both measurements are true ($\bar{ok}$ and ok).

This procedure is modeled in Figure 6. Note that the cat measurements in Figure 6 are not projective measurements (also called repeatable or von Neumann measurements) because they alter the state in the process of measurement. Specifically, $\bar{ok}$ evolves into ${\tilde{\psi }}_{4}$, and ok evolves into ${\tilde{\psi }}_{5}$. We are only really interested in the $\bar{fail}/\bar{ok}$ and fail/ok results, so this does not affect the discussion. It is staightforward to modify the circuit to perform a projective cat measurement using the technique of Figure 5 but it adds unnecessary complexity.

Not included are the agents’ reasoning circuits and, with them, their understanding of their statement qbits $S_{\bar{F}}$ and $S_{F}$. If the agents are reasoning correctly, statement $S_{\bar{F}}$ might reference the knowledge projection $|\psi_{2}^{\prime }\rangle$: “*I have observed t. The current state is $|\psi_{2}\rangle$. If the state were instead $|\psi_{2}^{\prime }\rangle$, then W’s measurement would result in ‘fail’*”. Note that knowledge projection is synonymous with *branch*, so the statement could be “*In my branch, W’s measurement will result in ‘fail’*”. We avoid this syntax because it is fairly misleading. She could also reason the following: “*as one component of a superposition, I have observed t. When W performs his physical measurement, he will observe ‘fail’ with a probability that depends on the amplitudes of the superposition*”. Statement $S_{F}$ could be the following: “*I have observed ↑. The current state is $|\psi_{3}\rangle$. If the state were instead $|\psi_{3}^{\prime }\rangle$, then $\bar{F}$ has recorded her belief in $S_{\bar{F}}$, which says that if the state were instead $|\psi_{2}^{\prime }\rangle$, then W’s measurement would result in ‘fail’*”.

The agent statements in FR are insufficiently qualified. For example, in the branch where $\bar{F}$ observes *t*, she states without qualification, “*I am certain that W will observe w= fail at time n:31*”. $\bar{F}$ fell victim to the virtual promotion fallacy. FR goes on to show that, in fact, *W* can measure “*ok*” in some runs, thus proving $\bar{F}$’s statement to be false. They do not address this discrepancy directly. Instead, they use $\bar{F}$’s statement in a chain of deduction using (Q), (C), and (S) to arrive at a contradiction. They then describe which of these deduction rules are violated in each of the various interpretations of quantum mechanics. In fact, there is nothing wrong with (Q), (C), and (S). (Q) is correct when applied to a state rather than a component of a superposition. (C) and (S) are correct when their premises are correct. It is $\bar{F}$’s false statement that causes the contradiction. On the other hand, the agent statements derived from our methodology are both true and consistent.

## 10. Weak Measurements

Non-invasive probes may be of use in WF scenarios by providing outside observers, such as W, with information about the state inside the laboratory with minimal disturbance. Such so-called weak measurements [23,24,25] can probe an observable complementary to that which is measured in the main experiment. In order to keep the disturbance small and respect uncertainty relations, the probe states for the weak measurement should have significant overlap, and additional statistics are necessary to obtain meaningful results. We can model a weak measurement in a quantum circuit as a controlled rotation of a probe qbit. If the rotation (probe interaction angle) is small, the probe overlap corresponding to different system states will be large, and the disturbance will be minimal.

**Figure 7:**
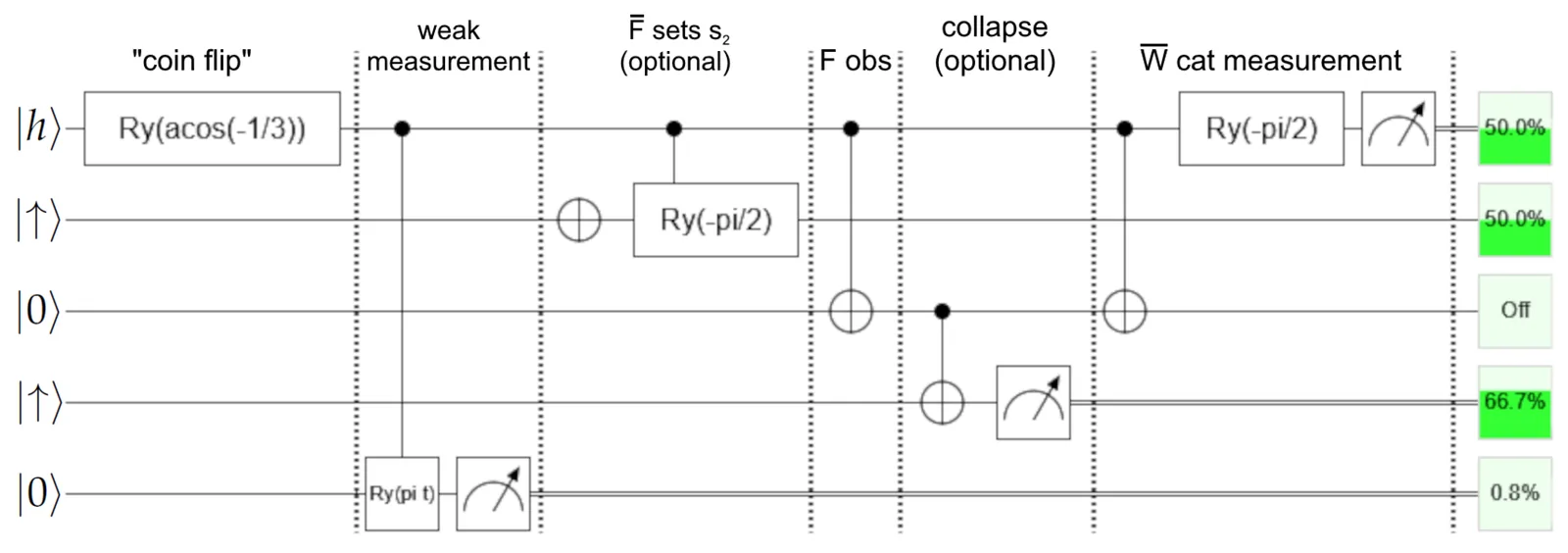
Weak measurement. The first three qbits are the same as in Figure 6. The new qbits are a probe for triggering a global collapse on $\bar{F}$’s knowledge and the weak probe.

Matzkin and Sokolovski (MS) have described several versions of weak measurements applied at various stages of the FR thought experiment [9]. In Figure 7, we model a version from Section III.B.1 of their paper. This version includes only observers $\bar{F}$ and $\bar{W}$. The spin set by $\bar{F}$ and observed by *F* is left out by MS, as it plays no role in the discussion. It does, however, modify the probabilities for $\bar{W}$’s cat measurement. This can be understood as resulting from summing over the degrees of freedom of the altered spin. We choose to include it as optional to give the reader the opportunity to experiment with it if desired. Our weak measurement operates in the $\sigma_{x}$ basis, which is the same basis that $\bar{F}$ will subsequently observe. The interaction is not the same as in MS, but this does not affect the discussion. The probe’s interaction angle is time-dependent, so users of the Quirk software can see the effect of different couplings in real time. $\bar{F}$’s observation includes an optional indirect measurement, which, if present, triggers global collapse. The collapse affects, but does not disrupt, $\bar{W}$’s cat measurement.

The probabilities that $\bar{W}$’s cat measurements result in $\bar{ok}$ are listed for various parameters in Table 2. When collapse is triggered, $\bar{F}$’s observation is a measurement, and $\bar{W}$’s probability is 1/2. Similarly, a probe angle of π corresponds to a maximal coupling, and $\bar{W}$ can determine what $\bar{F}$’s observation will be. His probability will, again, be 1/2. The probabilities for small angles reduce to the results without a weak measurement. For the case where $s_{2}$ is not present, the values in the table are in agreement with those derivable from Equations (26) and (28) of MS with small and large coupling λ.

If $s_{2}$ is not present and the probe angle is small, $\bar{W}$’s probability approaches ${\left(1-\sqrt{2}\right)}^{2}/6$. Thus, this experiment, like Deutsch’s, can determine if collapse occurred. The weak measurement is something of a red herring for this conclusion since the effect occurs even if the probe angle is zero.

MS go on to conclude that a spin state in superposition is inconsistent with $\bar{F}$ obtaining a definite outcome. We agree with this conclusion but disagree with the subsequent conclusion that WF leads to a contradiction.

## 11. Discussion

There is one metaphysical assumption in common among the various no-go theorems. It is sometimes called the Absoluteness of Observed Events (AOEs). One definition of this assumption is “an observed event is a real single event, and not relative to anything or anyone” [12]. There is a subtle additional implicit assumption that in each branch, the observer will make an unqualified statement of fact regarding their observation result. It is this implicit assumption that is wrong, and this loophole allows quantum theory to evade the no-go theorems. Our hypothetical agents are at least as intelligent as we are, would recognize they are in superposition, and would qualify their statements accordingly, perhaps using our definitions or some other way.

Perhaps the easiest way to lose one’s way with quantum observers is to treat virtual knowledge as absolute. This is understandable, as physicists are trained from the first few lessons of quantum mechanics to apply a projection operator during measurement. It is natural, but wrong, to extend this intuition to the case where an agent is entangled with the system but has not completed a measurement. Paraphrasing Peres [26], observations that are not measurements do not have absolute results.

## 12. Epilogue

Imagine you wake up in a laboratory with no memories. You are told you are a sophisticated quantum computer named F, and the entire laboratory evolves unitarily. You are handed a spin, a set of classical bits describing the experimental setup, and a virtual envelope containing classical bits representing the amplitudes of the up and down components of the spin. You read the setup details. You are asked to predict the probability that the experimenter W will later measure “up” but not to tell anyone. You are told to either observe the spin or open the envelope. After you have made your deduction, your Hamiltonian will be reversed, you will lose your memory, and you will be put back to sleep.

If you open the envelope, it will be easy to make the correct deduction. If, instead, you observe the spin as “up”, you still know you are in superposition, and you would realize you also need to know the relative amplitude of the other version of you since the experimenter will be measuring the entire quantum state, not just your current branch.

On the other hand, if you use our intuition (here, “you” represents you as a quantum AI, while “our intuition” means the author’s and the reader’s intuition. Please forgive any presumption) from basic quantum mechanics class, that any observation puts the system into an eigenstate of an observable (or, perhaps equivalently, that all observed events are absolute), then you would deduce, incorrectly, that the experimenter would definitely see “up”. Meanwhile, your counterpart would predict the opposite. Extrapolating our basic intuition to quantum observers is wrong and is at the core of the many paradoxes that arise out of Wigner’s Friend scenarios.

## Figures and Tables

**Table 1:** Experimental states relevant to FR. Each ψ is a vector in the 4 qbit Hilbert space. |−〉≡|0〉 indicates the agent records no statement, while $|S_{\bar{F}}\rangle \equiv |1\rangle$ or $|S_{F}\rangle \equiv |1\rangle$ indicate the respective agent has recorded her statement.

$|\psi_{0}\rangle$	$=\left(|h\;-↑-\rangle +\sqrt{2}|t\;-↑-\rangle \right)/\sqrt{3}$
$|\psi_{1}\rangle$	$=\left(|h\;-↓-\rangle +|t\;-↓-\rangle +|t\;-↑-\rangle \right)/\sqrt{3}$
$|\psi_{2}\rangle$	$=\left(|h\;-↓-\rangle +|t\;S_{\bar{F}}↓-\rangle +|t\;S_{\bar{F}}↑-\rangle \right)/\sqrt{3}$
$|\psi_{2}^{\prime }\rangle$	$=\left(|t\;S_{\bar{F}}↓-\rangle +|t\;S_{\bar{F}}↑-\rangle \right)/\sqrt{2}$
$|\psi_{3}\rangle$	$=\left(|h\;-↓-\rangle +|t\;S_{\bar{F}}↓-\rangle +|t\;S_{\bar{F}}↑S_{F}\rangle \right)/\sqrt{3}$
	$=\left(2|\bar{fail}↓-\rangle +|\bar{fail}↑S_{F}\rangle -|\bar{ok}↑S_{F}\rangle \right)/\sqrt{6}$
$|\psi_{3}^{\prime }\rangle$	$=|t\;S_{\bar{F}}↑S_{F}\rangle$
$|\psi_{4}\rangle$	$=-|\bar{ok}↑S_{F}\rangle$
	$=\left(|\bar{ok}\;ok\rangle -|\bar{ok}\;fail\rangle \right)/\sqrt{2}$
$|{\tilde{\psi }}_{4}\rangle$	$=|t\;-↑S_{F}\rangle$
$|\psi_{5}\rangle$	$=|\bar{ok}\;ok\rangle$
$|{\tilde{\psi }}_{5}\rangle$	=|t−↓−〉
|ok〉	$=\left(|↓-\rangle -|↑S_{F}\rangle \right)/\sqrt{2}$
|fail〉	$=\left(|↓-\rangle +|↑S_{F}\rangle \right)/\sqrt{2}$
$|\bar{ok}\rangle$	$=\left(|h\;-\rangle -|t\;S_{\bar{F}}\rangle \right)/\sqrt{2}$
$|\bar{fail}\rangle$	$=\left(|h\;-\rangle +|t\;S_{\bar{F}}\rangle \right)/\sqrt{2}$

**Table 2:** The probability of $\bar{W}$ to measure $\bar{ok}$ for various parameters of weak measurements.

$s_{2}$ Present	Probe Interaction Angle	$\bar{F}$ Collapse	$P_{\bar{\mathrm{ok}}}$
any	any	yes	1/2
any	π	no	1/2
yes	small	no	1/6
no	small	no	${\left(1-\sqrt{2}\right)}^{2}/6$

## Data Availability

The original contributions presented in this study are included in the article. Further inquiries can be directed to the corresponding author.
